# Impact of in-hospital postoperative complications on quality of life up to 12 months after major abdominal surgery

**DOI:** 10.1093/bjs/znad167

**Published:** 2023-06-19

**Authors:** Candice L Downey, Jamie Bainbridge, David G Jayne, David M Meads

**Affiliations:** Leeds Institute of Medical Research at St James’s, Clinical Sciences Building, St James’s University Hospital, University of Leeds, Leeds, UK; Academic Unit of Health Economics, School of Medicine, University of Leeds, Leeds, UK; Leeds Institute of Medical Research at St James’s, Clinical Sciences Building, St James’s University Hospital, University of Leeds, Leeds, UK; Academic Unit of Health Economics, School of Medicine, University of Leeds, Leeds, UK

## Abstract

**Background:**

Postoperative complications are common, but there are limited data regarding their implications on patients’ quality of life. This study aimed to address this gap in the literature by analysing the impact of postoperative complications on patients’ health-related quality of life.

**Methods:**

Data from the Perioperative Quality Improvement Programme were analysed, and included patient-level data for 19 685 adults who underwent elective major abdominal procedures in England since 2016. Postoperative complications were graded using the Clavien–Dindo classification. Quality of life was assessed by responses to the EuroQol five-dimension five-levels-of-response (EQ-5D-5L™) questionnaire before surgery, and at 6 and 12 months after operation. Ordinal logistic regression was used to estimate the association between Clavien–Dindo grades and quality of life. Tobit and ordinary least squares regression analyses were used to estimate the quality-adjusted life-year (QALY) loss resulting from postoperative complications between admission and 12 months after surgery.

**Results:**

At 6 and 12 months after surgery, increasingly severe postoperative complications were significantly associated with poorer health-related quality of life. The effect of postoperative complications on quality of life was sustained until at least 12 months after operation. Between admission and 12 months after surgery, 0.012, 0.026, 0.033, and 0.086 QALYs were lost for those experiencing a grade I, II, III, or IV postoperative complication respectively.

**Conclusion:**

Postoperative complications have a significant and sustained effect on patients’ quality of life after surgery; this effect worsens as the severity of the complications increases.

## Teaser

This work is the first to provide utility values for the different severities of postoperative complication, calculated from a large national data set of over 19 000 patients who had undergone major abdominal procedures across 124 UK hospitals. At 6 and 12 months after surgery, increasingly severe postoperative complications were shown to be significantly associated with poorer health-related quality of life. The effect of postoperative complications on quality of life was sustained until at least 12 months after surgery.

## Introduction

Every year the UK National Health Service (NHS) delivers more than 1.3 million general surgical procedures^1^. Up to 44 per cent of patients undergoing major surgery experience postoperative complications^2^, which can have a huge impact on patients and the healthcare system. A postoperative complication is defined as any deviation from the normal postoperative course^3^. A recent review^4^ of the literature found that postoperative complications contribute to increased mortality, longer duration of hospital stay, and an increased level of care at discharge. In the International Surgical Outcomes Study^5^, which collected data from 474 hospitals internationally in 2014, 2.8 per cent of patients who developed a postoperative complication died before discharge from hospital. This rises to 5–10 per cent for major surgical procedures^6^. The occurrence of a postoperative complication has been found to be more important than preoperative risk factors and intraoperative events in determining survival after major surgery^7^. For those who survive, complications also have adverse effects on long-term social functioning and quality of life^8^. As such, the prevention of postoperative complications is a priority for patients, clinicians, and healthcare systems.

In a healthcare system with fixed resources, new interventions must be shown to be cost-effective before they can be adopted widely. The National Institute for Health and Care Excellence (NICE) requires that the calculation of cost-effectiveness be based on the quality-adjusted life-year (QALY); this is a composite endpoint including quality-of-life assessment^9^. This approach allows comparisons between technologies and therapeutic areas so that the most impactful interventions can be chosen^10^. Communicating the potential impact of surgery on a patient’s quality of life can also inform shared decision-making before undertaking high-risk operations.

There are, however, limited data available regarding the implications of postoperative complications on patients’ quality of life. A Swedish study^11^ assessed the impact of severe complications (defined as Clavien–Dindo at least IIIb) on 1403 patients, 2 years after Roux-en-Y gastric bypass. Patients who had experienced a severe complication had a lower physical quality of life throughout the study, compared with matched controls. Another retrospective cohort study^12^ of 977 patients who had undergone reconstructive pelvic surgery found that higher Clavien–Dindo grades were associated with lower Short Form 36 scores, but patients were followed up for only 3 months.

There have been no large-scale research studies investigating the association between postoperative complications and quality of life. This study aimed to evaluate the effect of postoperative complications on patients’ quality of life after surgery, to determine whether there are other factors that influence quality of life after surgical complications and to estimate the disutility associated with different severities of postoperative complications, thereby enabling robust cost–utility analyses of interventions in future surgical research.

## Methods

Ethical approval for the study was granted by the University of Leeds School of Medicine Research Ethics Committee on 26 July 2022 (reference MREC 21-070). The Perioperative Quality Improvement Programme (PQIP) received ethics approval from the UK Health Research Authority (South-East Coast—Surrey Research Ethics Committee; REC reference 16/LO/1827). All patients gave informed consent to participate in PQIP. An anonymized data set was used for analysis.

This was a retrospective analysis of the PQIP database. PQIP is a national audit of more than 30 000 patients who have undergone selected elective major operations in 124 NHS hospitals since December 2016^13^. Patients are invited to participate in the study before admission to hospital, and consent to the collection of routine data about their care, and the completion of quality-of-life questionnaires before and after surgery. This national initiative aims to measure complications after major elective surgery and improve outcomes through feedback of data to clinicians.

The PQIP includes both Clavien–Dindo grades of postoperative complications and the EuroQol five-dimension five-levels-of-response (EQ-5D-5L™; EuroQol Group, Rotterdam, the Netherlands) scores for patients up to 12 months after surgery. The Clavien–Dindo classification defines complications as any deviation from the normal postoperative course and grades them according to severity^3^. It is an ordinal ranking system that assigns a grade to each postoperative complication based on the resources required for its treatment (*Table S1*). It has since become standard practice to classify general surgical complications using this system^14^. The EQ-5D-5L™ tool is one of the most extensively used multiattribute utility instruments for assessing health-related quality of life, measuring health across five dimensions: mobility, self-care, usual activities, pain and discomfort, and anxiety and depression. Each dimension has five possible responses which indicate progressively worse outcomes. These responses are converted into health state utilities which range from 1 (full health) to 0 (dead) to negative scores, which indicate health states considered to be worse than death.

### Patient population

Patients participating in PQIP who underwent major abdominal surgery were eligible for inclusion. Patients were included from across the 124 participating hospitals. Patients aged less than 18 years were excluded, as were those for whom a Clavien–Dindo grade was not recorded. Qualifying major abdominal procedures (determined by PQIP) are listed in the *supplementary material*, and included all major colorectal, hepatobiliary, and upper gastrointestinal operations.

### Data

Data of interest included baseline patient characteristics and clinical diagnoses, and the EQ-5D-5L™ dimension responses at 6 and 12 months after operation. Following NICE’s recommendation, EQ-5D-5L™ responses were mapped to the EQ-5D-3L™ index value using an existing algorithm^15^. Where EQ-5D™ responses were not available owing to the patient’s death, assumptions were made regarding the timing of death: individuals identified as having died between 0 and 6 months after operation were assumed to have died at 3 months, whereas those identified as having died between 6 and12 months after surgery were assumed to have died at 9 months. Patients who experienced a grade V complication were assumed to have died on admission.

Clavien–Dindo grades were condensed into five groups by combining subgrades IIIa and IIIb into a collective grade III, and subgrades IVa and IVb into a collective grade IV. This was to improve the ability to detect statistically significant associations in less frequent complications. In accordance with the PQIP data set, the worst complication experienced by the patient (that with the highest Clavien–Dindo grade) was used in the analysis.

A generalized ordinal logistic model was used to identify explanatory variables with a significant association with the likelihood of experiencing certain Clavien–Dindo grades. Adapting the ordinal logistic model into a generalized ordinal logistic model was required because of the violation of the proportional odds assumption^16^. Demographic variables included in the final model are listed in *Table 1*.

**Table 1:** Patient demographics

	Overall (*n* = 19 685)	Grade 0 (*n* = 10 737)	Grade I (*n* = 3162)	Grade II (*n* = 3646)	Grade III (*n* = 1386)	Grade IV (*n* = 541)	Grade V (*n* = 163)
Age (years), mean(s.d.)	63.8(13.7)	63.3(13.9)	63.8(14.2)	64.6(13.4)	63.9(13.2)	66.0(11.9)	72.3(9.8)
Sex ratio (F : M)	8335 : 11 350	4836 : 5901	1347 : 1815	1434 : 2212	494 : 892	158 : 383	46 : 117
BMI (kg/m^2^), mean(s.d.)	27.8(7.7)	27.6(6.8)	27.7(7.0)	28.1(10.8)	27.7(5.6)	28.0(8.0)	27.5(6.4)
**ASA physical status grade**							
I	1814 (9.2)	1221 (11.4)	272 (8.6)	211 (5.8)	79 (5.7)	22 (4.1)	5 (3.1)
II	12 255 (62.3)	6902 (64.3)	1952 (61.7)	2148 (58.9)	854 (61.6)	308 (56.9)	66 (40.5)
III	5422 (27.6)	2531 (23.6)	905 (28.6)	1242 (34.1)	438 (31.6)	203 (37.5)	86 (52.8)
IV	181 (0.9)	77 (0.7)	33 (1.0)	41 (1.1)	14 (1.0)	8 (1.5)	5 (3.1)
V	10 (0.05)	5 (0.1)	0 (0)	3 (0.1)	1 (0.1)	0 (0)	1 (0.6)
**ECG findings**							
No abnormalities	13 869 (77.1)	7642 (78.4)	2172 (76.2)	2556 (75.5)	980 (75.9)	377 (73.9)	102 (67.6)
AF rate 60–90	697 (3.9)	356 (3.7)	125 (4.4)	133 (3.9)	42 (3.3)	27 (5.3)	13 (8.6)
AF rate > 90*	2129 (11.8)	1022 (10.5)	347 (12.2)	481 (14.2)	161 (12.5)	82 (16.1)	31 (20.5)
Not done	1292 (7.2)	732 (7.5)	206 (7.2)	214 (6.3)	109 (8.4)	24 (4.7)	5 (3.3)
**Cardiac history**							
No	15 076 (76.6)	8612 (80.2)	2353 (74.4)	2609 (71.6)	1026 (74.0)	348 (64.3)	89 (54.6)
Yes	4607 (23.4)	2124 (19.8)	809 (25.6)	1036 (28.4)	360 (26.0)	193 (35.7)	74 (45.4)
**Heart function class**†							
I	16 284 (82.7)	9068 (84.5)	2580 (81.6)	2974 (81.6)	1116 (80.5)	396 (73.2)	108 (66.3)
II	2866 (14.6)	1419 (13.2)	496 (15.7)	559 (15.3)	236 (17.0)	111 (20.5)	40 (24.5)
III	515 (2.6)	242 (2.3)	84 (2.7)	108 (3.0)	33 (2.4)	33 (6.1)	14 (8.6)
IV	15 (0.1)	6 (0.1)	2 (0.1)	4 (0.1)	1 (0.1)	1 (0.2)	1 (0.6)
**Respiratory findings**							
No dyspnoea	15 505 (86.2)	8649 (88.7)	2418 (84.8)	2806 (82.9)	1085 (84.0)	405 (79.4)	99 (65.6)
Dyspnoea at rest	21 (0.1)	9 (0.1)	1 (0)	5 (0.2)	2 (0.2)	2 (0.4)	2 (1.3)
Dyspnoea limiting exertion	467 (2.6)	189 (1.9)	91 (3.2)	107 (3.2)	38 (2.9)	26 (5.1)	15 (9.9)
Dyspnoea on exertion	2001 (11.1)	907 (9.3)	343 (12.0)	466 (13.8)	167 (12.9)	77 (15.1)	35 (23.2)
**Liver disease**							
None	17 820 (98.8)	9689 (99.1)	2828 (98.8)	3341 (98.7)	1268 (98.0)	500 (97.7)	146 (96.1)
Cirrhosis or hepatitis B/C with portal HTN	38 (0.2)	16 (0.2)	5 (0.2)	9 (0.3)	2 (0.2)	3 (0.6)	2 (1.3)
Cirrhosis or hepatitis B/C without portal HTN	180 (1.0)	76 (0.8)	30 (1.1)	36 (1.1)	24 (1.9)	9 (1.8)	4 (2.6)
**Smoking history**							
Never smoked	9597 (48.8)	5417 (50.5)	1583 (50.1)	1707 (46.8)	616 (44.4)	190 (35.1)	66 (40.5)
Current smoker	1964 (10.0)	1028 (9.6)	322 (10.2)	377 (10.3)	140 (10.1)	78 (14.4)	14 (8.6)
Ex-smoker quit > 6 months ago	6494 (33.0)	3420 (31.9)	974 (30.8)	1290 (35.4)	506 (36.5)	213 (39.4)	67 (41.1)
Ex-smoker quit ≤ 6 months ago	833 (4.2)	389 (3.6)	142 (4.5)	171 (4.7)	74 (5.3)	44 (8.1)	11 (6.8)
Unknown	796 (4.0)	483 (4.5)	141 (4.5)	100 (2.7)	50 (3.6)	16 (3.0)	5 (3.1)
**Alcohol consumption (units/day)**							
None	6611 (36.8)	3424 (35.1)	1042 (36.6)	1370 (40.5)	518 (40.2)	180 (35.2)	64 (42.4)
0–2	7502 (41.7)	4198 (43.0)	1189 (41.7)	1326 (39.2)	484 (37.5)	219 (42.9)	54 (35.8)
3–4	1732 (9.6)	964 (9.9)	247 (8.7)	333 (9.8)	117 (9.1)	52 (10.2)	15 (10.0)
> 5	875 (4.9)	442 (4.5)	160 (5.6)	151 (4.5)	81 (6.3)	32 (6.3)	9 (6.0)
Unknown	1271 (7.1)	727 (7.5)	213 (7.5)	203 (6.0)	90 (7.0)	28 (5.5)	9 (6.0)
**Diabetes**							
No	16 967 (86.2)	9407 (87.6)	2695 (85.2)	3076 (84.4)	1187 (85.6)	431 (79.7)	131 (80.4)
Type I	121 (0.6)	60 (0.6)	20 (0.6)	22 (0.6)	11 (0.8)	7 (1.3)	1 (0.6)
Type II—diet controlled only	609 (3.1)	302 (2.8)	104 (3.3)	119 (3.3)	51 (3.7)	19 (3.5)	9 (5.5)
Type II—on insulin	554 (2.8)	269 (2.5)	99 (3.1)	117 (3.2)	34 (2.5)	25 (4.6)	7 (4.3)
Type II—non-insulin medication	1432 (7.3)	698 (6.5)	244 (7.7)	311 (8.5)	103 (7.4)	59 (10.9)	15 (9.2)
**Planned postprocedure destination**							
Ward care	8447 (42.9)	5386 (50.2)	1414 (44.7)	1093 (30.0)	425 (30.7)	92 (17.0)	29 (17.8)
Level 1 or enhanced care	2790 (14.2)	1642 (15.3)	455 (14.4)	473 (13.0)	171 (12.3)	31 (5.7)	14 (8.6)
Level 2 care	7065 (35.9)	3335 (31.1)	1128 (35.7)	1615 (44.3)	620 (44.7)	257 (47.5)	83 (50.9)
Level 3 care	1376 (7.0)	372 (3.5)	164 (5.2)	463 (12.7)	170 (12.3)	161 (29.8)	37 (22.7)
**Perioperative risk assessment**							
Qualitative	5198 (26.4)	2921 (27.2)	823 (26.0)	960 (26.3)	313 (22.6)	131 (24.2)	40 (24.5)
Quantitative	4047 (20.6)	2016 (18.8)	680 (21.5)	803 (22.0)	352 (25.4)	148 (27.4)	34 (20.9)
Both	4093 (20.8)	2257 (21.0)	633 (20.0)	731 (20.1)	298 (21.5)	122 (22.6)	44 (27.0)
None	6343 (32.2)	3542 (33.0)	1025 (32.4)	1150 (31.6)	423 (30.5)	140 (15.9)	45 (27.6)
**Planned surgical specialty**							
Hepatobiliary	2316 (11.8)	1117 (10.4)	331 (10.5)	559 (15.3)	201 (14.5)	75 (13.9)	31 (19.0)
Lower gastrointestinal	14 274 (72.5)	8229 (76.6)	2443 (77.3)	2327 (63.8)	874 (63.1)	279 (51.6)	94 (57.7)
Upper gastrointestinal	2173 (11.0)	904 (8.4)	250 (7.9)	567 (15.6)	236 (17.0)	168 (31.1)	32 (19.6)
Other	922 (4.7)	487 (4.5)	138 (4.4)	193 (5.3)	75 (5.4)	19 (3.5)	6 (3.7)
**Cancer diagnosis**							
Yes	7212 (75.2)	3881 (74.4)	1217 (74.6)	1406 (77.2)	468 (74.8)	182 (80.5)	57 (89.1)
No	2378 (24.8)	1339 (25.6)	414 (25.4)	416 (22.8)	158 (25.2)	44 (19.5)	7 (10.9)
**Operations in past 30 days**							
1	19 012 (96.6)	10 442 (97.3)	3045 (96.3)	3491 (95.8)	1318 (95.1)	513 (94.8)	157 (96.3)
> 1	668 (3.4)	293 (2.7)	117 (3.7)	154 (4.2)	68 (4.9)	28 (5.2)	6 (3.7)
**Urgency of surgery**							
Elective	18 134 (92.1)	9915 (92.3)	2869 (90.7)	3384 (92.8)	1276 (92.1)	499 (92.2)	146 (89.6)
Expedited	1551 (7.9)	822 (7.7)	293 (9.3)	262 (7.2)	110 (7.9)	42 (7.8)	17 (10.4)
**Employment status**							
Employed	6175 (31.4)	3561 (33.2)	1016 (32.1)	1027 (28.2)	415 (30.0)	127 (23.5)	17 (10.4)
Parent or carer	413 (2.1)	253 (2.4)	63 (2.0)	63 (1.7)	20 (1.4)	11 (2.0)	2 (1.2)
Retired	10 253 (52.1)	5439 (50.7)	1628 (51.5)	1987 (54.5)	728 (52.5)	325 (60.1)	117 (71.8)
Unavailable	1405 (7.1)	791 (7.4)	224 (7.1)	256 (7.0)	82 (5.9)	32 (5.9)	19 (11.7)
Unemployed	342 (1.7)	178 (1.7)	60 (1.9)	67 (1.8)	29 (2.1)	5 (0.9)	1 (0.6)
Unemployed owing to health	1097 (5.6)	515 (4.8)	171 (5.4)	246 (6.8)	112 (8.1)	41 (7.6)	7 (4.3)

Values are *n* (%) unless indicated otherwise. Clavien–Dindo grade was missing for 50 individuals. Missing data may result in the sum of data entries not equalling the corresponding number of indivudals in each grade. *Includes any other abnormal rhythm, paced rhythm, more than 5 VE per min, Q ST or T wave abnormalities. †New York Heart Association functional classification. AF, atrial fibrillation; HTN, hypertension; VE, ventricular ectopics.

### Analytical methods

The association between postoperative complications and quality of life was estimated using EQ-5D-5L™ data in three ways. First, the association between Clavien–Dindo grades and individual EQ-5D-5L™ dimension responses was examined using ordinal logistic regression. Second, mean EQ-5D-3L™ index values for each Clavien–Dindo grade were plotted across three time points: admission, and 6 and 12 months after surgery. Finally, the area-under-the-curve method was used to calculate individual-level QALYs by summing the areas of the geometrical shapes obtained by linearly interpolating between EQ-5D-3L™ index values at admission, and 6 and 12 months after operation^17^, before Tobit and ordinary least squares regressions were used to estimate the QALY loss owing to postoperative complications between admission and 12 months after surgery. Admission EQ-5D-3L™ index values were included to control for differences in baseline health^17^.

To estimate the QALY loss to the NHS in England in 2018–2019, an estimated volume of major abdominal procedures was required. To calculate this volume, a predicted 1.3 million general surgical procedures in 2013–2014 provided a reference point^18^. In 2013–2014, the proportion of surgical procedures classified as major was estimated at 19.5 per cent^19^. By applying procedure growth rates from this study, the estimated number of NHS major abdominal surgical procedures in England in 2018–2019 was 269 348. Grade I–IV multivariable ordinary least squares coefficients were then multiplied by the estimated frequency of grades I–IV complications to generate an estimated QALY loss.

When accounting for missing data, complete-case analysis was deemed appropriate for the ordinal logistic regression which identified the predictors of postoperative complications. In regression analyses that included the QALYs variable, multiple imputation was used to increase the sample size from 8693 to 16 514. The missingness mechanism for the QALYs variable was assumed not to be missing not at random and, therefore, not in violation of the requirements for multiple imputation. Missing values were estimated to generate 20 imputed data sets before the results from each data set were pooled^18^.

All statistical analyses were performed using Stata^®^ software version 17.0 (StataCorp, College Station, TX, USA). All statistical tests were two-sided and, unless stated otherwise, statistical significance was determined based on an α of 0.05.

## Results

### Patient demographics

Individual-level data for 19 685 patients who underwent qualifying elective major abdominal procedures were analysed. Procedures were categorized into lower gastrointestinal (72.5 per cent), hepatobiliary (11.8 per cent), lower gastrointestinal (11.0 per cent), and other (4.7 per cent). The mean age of the included patients was 63.8 years. Men constituted 57.7 per cent of the population. Most of the patients had an ASA grade of II (62.3 per cent). Some 75.2 per cent of participants had an active cancer diagnosis at the time of surgery. A summary of baseline patient characteristics for the full cohort, and by Clavien–Dindo grade, is presented in *Table 1*.

### Predictors of complications

The regression sample included 17 900 individuals who had data on Clavien–Dindo grade and a full set of explanatory variables. The impact of each demographic variable on the probability of experiencing each level of Clavien–Dindo grade is shown in *Table S2*.

Besides postoperative destination, the largest prognostic drivers of experiencing any postoperative complication (Clavien–Dindo grade higher than 0 *versus* 0 (grade 0 is the lowest CD grade)) were: dyspnoea limiting exertion or dyspnoea on rest *versus* no dyspnoea; having a cardiac history *versus* no cardiac history; and being unemployed for health reasons *versus* being employed. Regarding fatal complications (grade over IV *versus* IV or less), the largest prognostic drivers were being male and having any respiratory diagnosis.

Age was not a significant predictor of the severity of postoperative complications until Clavien–Dindo grades III and above. A 1-year increase in age increased the probability of experiencing a complication with a Clavien–Dindo grade of more than III *versus* III or less by 1.5 per cent (*P* < 0.001), and the probability of experiencing a complication of grade higher than IV *versus* IV or less by 6.0 per cent (*P* < 0.001).

A recent history of smoking had more effect on the likelihood of experiencing severe complications after surgery. Compared with an individual who had never smoked, an individual classified as ‘ex-smoker—stopped 6 months ago or less’, had a 30.5 per cent increase in probability of experiencing a complication of grade higher than IV *versus* IV or less (*P* < 0.001).

### Postoperative complications and quality of life

Of the 19 685 participants, 34 per cent had complete EQ-5D-5L™ at all 3 time points (admission, 6 months, and 12 months). At admission, 91.8 per cent of patients had complete quality-of-life data; this dropped to 52 per cent at 6 months after surgery, and 45 per cent at 12 months (*Tables S3* and *S4*). Univariate logistic regression analysis showed that different grades of complication were not significantly associated with the likelihood of having missing EQ-5D™ data, supporting the use of multiple imputation to account for the missing data.

At both 6 and 12 months after surgery, Clavien–Dindo grades of higher severity were associated with an increased likelihood of the patient selecting a higher-severity EQ-5D-5L™ response. In particular, the dimensions of self-care and usual activities were affected; for example, at 6 months after surgery, individuals who experienced a grade IV postoperative complication were 2.47 times more likely to select a response indicating a greater impact on usual activities than individuals who did not experience a postoperative complication (*P* < 0.001). For grades III and IV complications, every one of the five dimension coefficients had increased at 6 and 12 months after surgery compared with at admission.

At 6 months after surgery, experiencing any postoperative complication was significantly associated with an increased likelihood of selecting a higher-severity EQ-5D™ response for any dimension. At 12 months, experiencing a grade II, III, or IV complication was significantly associated with an increased likelihood of selecting a higher-severity EQ-5D-5L™ response for any dimension.

### Quality-of-life trajectory

**Fig. 1:**
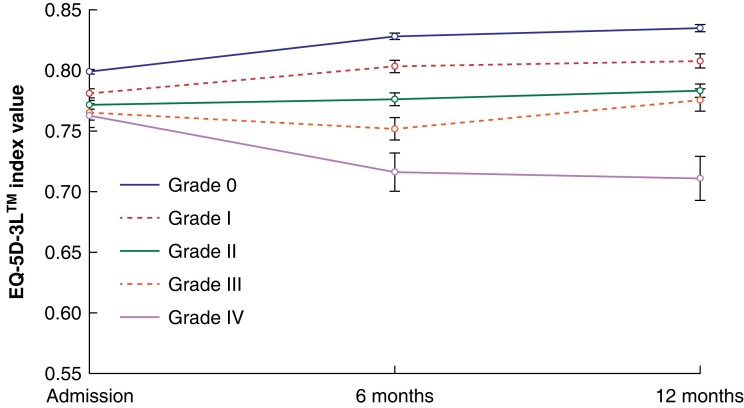
Quality-of-life trajectory for each Clavien–Dindo grade using mean EQ-5D-5L™ values Error bars represent 95% confidence intervals.

**Fig. 2:**
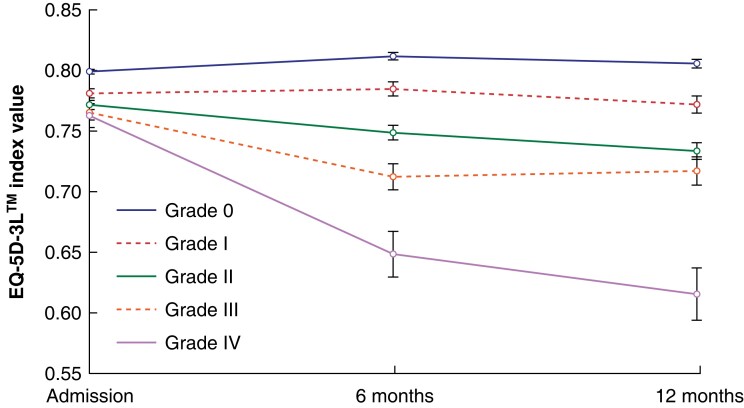
Quality-of-life trajectory for each Clavien–Dindo grade using mean EQ-5D-5L™ values adjusted for mortality Error bars represent 95% confidence intervals.

**Fig. 3:**
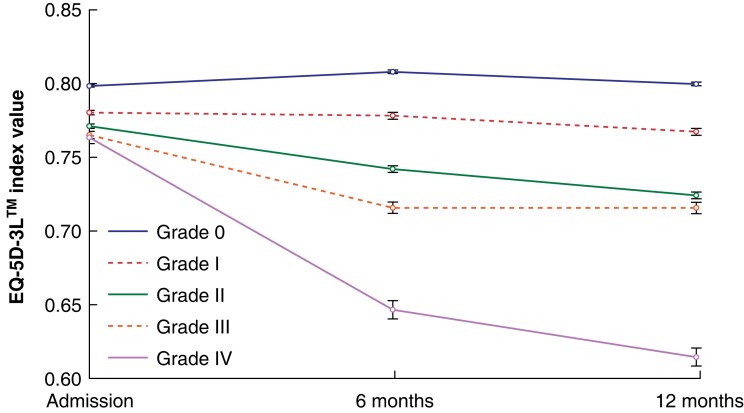
Quality-of-life trajectory for each Clavien–Dindo grade using mean EQ-5D-3L™ values adjusted for mortality and missing data Error bars represent 95% confidence intervals. Multiple imputation was used in analysis of 6- and 12-month data.

Mean EQ-5D™ utility index values for each complication grade were plotted between admission, 6 months, and 12 months after surgery (*Figs 1–3*). At admission, mean EQ-5D™ values were lower in patients who went on to experience a higher grade of postoperative complication, indicating that individuals who experience more severe postoperative complications are more likely to have poorer health before admission.

Mean EQ-5D™ values increased between admission and 12 months after surgery for all patients, except those who experienced a grade IV complication, for whom it decreased by 0.052 (*Fig. 1*).

Mortality was accounted for by assigning postoperative EQ-5D™ index values of 0 to individuals identified as deceased. When mortality data were included in the analysis, the mean EQ-5D™ values increased only for patients who did not experience a postoperative complication; Clavien–Dindo grades I, II, III, and IV complications were associated with decreases in mean EQ-5D™ values of 0.009, 0.039, 0.048, and 0.148 respectively (*Fig. 2*).

At 6 and 12 months after surgery, the response rate of individuals to the EQ-5D™ questionnaire had fallen from 91.8 per cent at admission, to 53.2 and 46.5 per cent respectively. When the analysis was adjusted for missing data, the same trends were seen (*Fig. 3*); only patients who did not experience a postoperative complication had an increase in EQ-5D values™. Clavien–Dindo grade I, II, III, and IV complications were associated with decreases in the mean EQ-5D™ values of 0.013, 0.047, 0.049, and 0.148 respectively.

### Quality-adjusted life-years

QALYs could be calculated for 44.9 per cent of individuals; multiple imputation was used to address missing data and increase the sample size from 8693 to 16 514. The regression results are available in *Table S5*.

Each Clavien–Dindo grade was significantly associated with decreasing QALYs, to at least 5 per cent significance. As the Clavien–Dindo grade increased, the magnitude of its impact on QALY loss increased. Between admission and 12 months after surgery, 0.012, 0.026, 0.033, and 0.086 QALYs were lost if an individual experienced a grade I, II, III, or IV postoperative complication respectively, relative to an individual who did not experience a complication.

An estimated 3083 QALYs were lost in the NHS in England in 2018–2019 during the 12 months after surgery (*Table S6*).

## Discussion

This study has shown that postoperative complications have a significant effect on patients’ quality of life after surgery; this effect worsens as the severity of the complications increases, and is sustained until at least 12 months after operation. The analysis estimated substantial QALY loss for each grade of complication: 0.012, 0.026, 0.033, and 0.086 QALYs for individuals experiencing a grade I, II, III, or IV postoperative complication respectively. Comparison of these utility values with those in other studies indicated that patients who experience a grade III or IV complication have poorer quality of life than people living with advanced colorectal cancer^20^. If QALYs are valued at £20 000 (€23 000) each, £61 680 000 (€70 870 000) of net benefit loss is incurred each year by the NHS owing to surgical complications in this population. This does not include the cost of healthcare resource use associated with complications.

A small number of studies estimating the association between postoperative complications and health-related quality of life have been published so far. These mostly involved small populations, diverse quality-of-life tools, and short follow-up times. A Swedish study^11^ found that patients who had a severe complication after Roux-en-Y gastric bypass had a lower physical quality of life even 2 years later. This supports the findings of the present study of a prolonged effect of postoperative complications. The study sample size and prolonged follow-up time has generated statistically credible findings. The outcomes are also more generalizable than current evidence, given that a range of major abdominal procedures was included in the population. The use of the EQ-5D-5L™ enhances the usefulness of the findings, as it is the measure preferred by NICE for use in economic evaluations. In the literature, only one study^21^ used a EuroQol tool; the EQ-5D-3L™ questionnaire was employed in a retrospective cohort study of 45 patients undergoing glioma surgery, and the authors reported that patients with new-onset neurological deficit had a deterioration in health-related quality of life at 3 months after surgery.

The present study is limited by the precision of the available data. This is particularly pertinent for the analyses involving mortality; the PQIP data set does not include dates of death and so assumptions were made to enable analysis. The actual length of time between admission and death for patients who experienced a grade V complication will determine the overestimation of their QALY loss. In contrast, the impact of any complications that occurred outside of the index admission could not be estimated because of limitations of the data set; this could have led to underestimation of the QALY loss associated with such complications.

A further limitation may have been the use of only the complication with the highest Clavien–Dindo grade in the analyses. This may have led to underestimation of the total burden of postoperative morbidity, especially in patients with multiple postoperative complications. Follow-up for PQIP study participants ends at 12 months after operation, and so the calculation of QALY losses was limited to this time interval. As the analyses indicated a sustained impact of postoperative complications on quality of life at 12 months after operation, it is reasonable to assume that further QALYs are lost in subsequent months owing to postoperative complications. Further research is required to allow inclusion of the estimated lifetime impact of these complications in the calculation of associated QALY losses.

To the authors’ knowledge, this is the first large-scale study of the impact of different Clavien–Dindo grades of complication on health-related quality of life after major abdominal surgery. The analysis has shown that postoperative complications have a sustained effect on patients’ quality of life; patients should be made aware of their potential impact before treatment. An estimated QALY loss provides a compelling case for investing in future research to predict, prevent, and manage postoperative complications. The quality-of-life implications estimated by this study can now be used to enable robust cost–utility analyses of interventions in such research. Further analyses of quality of life with longer time horizons will provide even more accurate data regarding the health economics of postoperative complications.

## Supplementary Material

znad167_Supplementary_Data

## Data Availability

The data sets used and/or analysed for the present study are not available owing to the limitations of PQIP ethical approvals. The analytical methods can be made available to other researchers on reasonable request to the corresponding author.
