# Supplementary material for: Impact of in-hospital postoperative complications on quality of life up to 12 months after major abdominal surgery
Source: Br J Surg. 2023 Jun 19;110(9):1206–12. doi: 10.1093/bjs/znad167 (PMC10416679; doi:10.1093/bjs/znad167)
Supplement: znad167_Supplementary_Data [file znad167_supplementary_data.docx]

**The impact of in-hospital postoperative complications on quality of life up to 12 months after major abdominal surgery**

Candice L Downey^1^, Jamie Bainbridge^2^, David G Jayne^1^, David M Meads^2^

1. Leeds Institute of Medical Research at St James’s, Clinical Sciences Building,
   St. James’s University Hospital, University of Leeds, Leeds, LS9 7TF
2. Academic Unit of Health Economics, School of Medicine, University of Leeds, Leeds, LS9 7TF

**Correspondence to:**

Miss Candice L Downey

Level 7, Clinical Sciences Building

St James’s University Hospital

Leeds

LS9 7TF

Email: [c.l.downey@leeds.ac.uk](mailto:c.l.downey@leeds.ac.uk)

ORCID: 0000-0001-9818-8002

Twitter: @MissCDowney

**Supplementary Materials - Index**

| **Supplementary Methods** |  |
| --- | --- |
| **Methods.** PQIP qualifying major abdominal procedures | Page 3 |
| **Supplementary Tables** |  |

| **Table S1.** The Clavien-Dindo Classification of Surgical Complications | Page 5 |
| --- | --- |
| **Table S2.** Results of the generalised ordinal logistic regression identifying the predictors of postoperative complications. | Page 6 |
| **Table S3.** Data completeness at three timepoints | Page 9 |
| **Table S4.** Patterns of EQ-5D-5L missingness | Page 9 |
| **Table S5.** Tobit, OLS, and multiple imputation regression results. | Page 10 |
| **Table S6.** English NHS morbidity QALY loss due to postoperative complications in 2018/2019. | Page 12 |

**Methods. PQIP qualifying major abdominal procedures**

**Lower gastrointestinal**

Abdominal operation for Hirschprung's disease
Abdominal revision of restorative proctocolectomy

Abdominoperineal (AP) resection with anastomosis (+/- pouch)
Abdominoperineal resection with end colostomy

Abdominoperineal pull-though resection with colo-anal anastomosis +/- colonic pouch and associated stoma

Anterior resection

Colectomy (total and ileorectal anastomosis)
Colectomy and colostomy and preservation of the rectum

Completion proctectomy

Excision of retroperitoneal Tumour (+/- Ureterolysis)
Excision of transverse colon
Exploratory laparotomy
Hartmann's procedure
Ileoanal anastomosis and creation of pouch
Ileo-caecal resection (with anastomosis or ileostomy formation)
Laparotomy for enterocutaneous fistula
Left hemicolectomy (with anastomosis /colostomy)

Panproctocolectomy and ileostomy
Partial excision of rectum and sigmoid colon for prolapse

Redo operations on ileum/colon
Resection of duodenal tumour
Resection of small bowel (+/- tumour)
Reversal of Hartmann's procedure
Right hemicolectomy (with anastomosis /colostomy)

Sigmoid colectomy

**Hepatobiliary**

Anastomosis of gall bladder (to another viscus)

Anastomosis of hepatic duct
Anastomosis of pancreatic duct (to another viscus)

Associating Liver Partition and Portal vein

Ligation for Staged hepatectomy (ALPPS)

Creation of portocaval shunt
Excision of lesion of bile duct
Excision of lesion – Pancreas

Exploratory laparotomy
Frey's procedure
Hepatectomy (Partial / Hemi/ Total)
Hemihepatectomy (right/left)
Pancreatectomy (partial/distal/total)

Pancreaticoduodenectomy (Whipple procedure)
Partial excision of bile duct and anastomosis of bile duct to duodenum and jejunum

Radiofrequency thermocoagulation of liver with scalpel liver resection
Repair of bile duct
Resection of lesion(s) of liver

Splenectomy (partial/total)

**Upper gastrointestinal**

Anastomosis of gall bladder (to another viscus)

Anastomosis of hepatic duct
Anastomosis of pancreatic duct (to another viscus)

Bypass of oesophagus

Closure of bypass of oesophagus
Endoscopically assisted oesophagectomy
Excision of lesion of pancreas / bile duct / liver
Exploratory laparotomy
Gastrectomy (partial / total) with excision of surrounding tissue
Hepatectomy including partial
Hemi hepatectomy (right/left)
Ileo-caecal resection (with anastomosis or ileostomy formation)
Laparoscopic biliary gastric bypass
Laparotomy for enterocutaneous fistula

Oesophagocardiomyotomy (Heller’s Procedure)

Oesophagectomy (partial /total)

Oesophagogastrectomy

Open excision of lesion of oesophagus
Pancreatectomy (partial/distal/total)

Pancreaticoduodenectomy (Whipple procedure)
Partial excision of bile duct and anastomosis of bile duct to duodenum/jejunum
Radiofrequency thermocoagulation of the liver with scalpel resection
Repair of bile duct

**Other**

Adrenalectomy (unilateral/bilateral)
Complex abdominal wall reconstruction
Complex restoration of intestinal continuity
Cytoreductive surgery +/- intraperitoneal chemotherapy

Excision of retroperitoneal tumour (+/- ureterolysis)

Exploratory laparotomy
Intestinal failure reconstruction
Laparotomy + excision of sarcoma tumour
Laparotomy + restoration of intestinal continuity
Pelvic exenteration
Retroperitoneal lymph node dissection

**Table S1. The Clavien-Dindo Classification of Surgical Complications**

| **Grade** | **Definition** |
| --- | --- |
| I | Any deviation from the normal postoperative course without the need for pharmacological, surgical, endoscopic or radiological interventions. Acceptable therapeutic regimens are: antiemetics, antipyretics, analgesics, diuretics, electrolytes, physiotherapy and wounds opened at the bedside. |
| II | Complication requiring pharmacological treatment with drugs other than those allowed for Grade I complications |
| IIIa | Complication requiring surgical, endoscopic or radiological intervention under regional or local anaesthesia |
| IIIb | Complication requiring surgical, endoscopic or radiological intervention under general anaesthesia |
| IVa | Life-threatening complication requiring critical care management of single organ dysfunction |
| IVb | Life-threatening complication requiring critical care management of multi-organ dysfunction |
| V | Death |

**Table S2. Results of the generalised ordinal logistic regression identifying the predictors of postoperative complications**

| Variable | Proportional odds upheld? | | Odds ratio CDC>0 vs <=0 (SD) | | 95% conf. interval | | Odds ratio CDC>I vs <=I (SD) | | 95% conf. interval | | Odds ratio CDC>II vs <=II (SD) | | 95% conf. interval | | Odds ratio CDC>III vs <=III (SD) | | 95% conf. interval | | Odds ratio CDC>IV vs <=IV (SD) | | 95% conf. interval | |  |
| --- | --- | --- | --- | --- | --- | --- | --- | --- | --- | --- | --- | --- | --- | --- | --- | --- | --- | --- | --- | --- | --- | --- | --- |
|  |  | |  | |  | |  | |  | |  | |  | |  | |  | |  | |  | |  |
| Age | No | | .999 (.002) | | [.996 - 1.002] | | 1.001 (.002) | | [.997 - 1.004] | | 1.001 (.002) | | [.996 - 1.005] | | 1.015*** (.004) | | [1.008 - 1.023] | | 1.060*** (.010) | | [1.042 - 1.079] | |  |
|  |  | |  | |  | |  | |  | |  | |  | |  | |  | |  | |  | |  |
| Gender |  | |  | |  | |  | |  | |  | |  | |  | |  | |  | |  | |  |
| Female | *Reference* | | - | | - | | - | | - | | - | | - | | - | | - | | - | | - | |  |
| Male | No | | 1.175*** (.038) | | [1.102 - 1.253] | | 1.207*** (.043) | | [1.125 - 1.295] | | 1.348*** (.071) | | [1.216 - 1.495] | | 1.550*** (.137) | | [1.303 - 1.844] | | 1.626*** (.296) | | [1.138 - 2.324] | |  |
|  |  | |  | |  | |  | |  | |  | |  | |  | |  | |  | |  | |  |
| BMI^1^ | Yes | | 1.001 (.002) | | [.997 - 1.004] | | 1.001 (.002) | | [.997 - 1.004] | | 1.001 (.002) | | [.997 - 1.004] | | 1.001 (.002) | | [.997 - 1.004] | | 1.001 (.002) | | [.997 - 1.004] | |  |
|  |  | |  | |  | |  | |  | |  | |  | |  | |  | |  | |  | |  |
| ASA^2^ |  | |  | |  | |  | |  | |  | |  | |  | |  | |  | |  | |  |
| Class I | Yes | | .721*** (.040) | | [.646 - .804] | | .721*** (.040) | | [.646 - .804] | | .721*** (.040) | | [.646 - .804] | | .721*** (.040) | | [.646 - .804] | | .721*** (.040) | | [.646 - .804] | |  |
| Class II | *Reference* | | - | | - | | - | | - | | - | | - | | - | | - | | - | | - | |  |
| Class III | Yes | | 1.152*** (.041) | | [1.074 - 1.236] | | 1.152*** (.041) | | [1.074 - 1.236] | | 1.152*** (.041) | | [1.074 - 1.236] | | 1.152*** (.041) | | [1.074 - 1.236] | | 1.152*** (.041) | | [1.074 - 1.236] | |  |
| Class IV or V | Yes | | 1.132 (.165) | | [.851 - 1.506] | | 1.132 (.165) | | [.851 - 1.506] | | 1.132 (.165) | | [.851 - 1.506] | | 1.132 (.165) | | [.851 - 1.506] | | 1.132 (.165) | | [.851 - 1.506] | |  |
|  |  | |  | |  | |  | |  | |  | |  | |  | |  | |  | |  | |  |
| ECG^3^ |  | |  | |  | |  | |  | |  | |  | |  | |  | |  | |  | |  |
| No abnormalities | *Reference* | | - | | - | | - | | - | | - | | - | | - | | - | | - | | - | |  |
| AF rate 60-90 | Yes | | .901 (.069) | | [.775 - 1.046] | | .901 (.069) | | [.775 - 1.046] | | .901 (.069) | | [.775 - 1.046] | | .901 (.069) | | [.775 - 1.046] | | .901 (.069) | | [.775 - 1.046] | |  |
| AF rate > 90^4^ | Yes | | 1.085* (.050) | | [.992 - 1.186] | | 1.085* (.050) | | [.992 - 1.186] | | 1.085* (.050) | | [.992 - 1.186] | | 1.085* (.050) | | [.992 - 1.186] | | 1.085* (.050) | | [.992 - 1.186] | |  |
| No investigation | Yes | | 1.074 (.063) | | [.958 - 1.205] | | 1.074 (.063) | | [.958 - 1.205] | | 1.074 (.063) | | [.958 - 1.205] | | 1.074 (.063) | | [.958 - 1.205] | | 1.074 (.063) | | [.958 - 1.205] | |  |
|  |  | |  | |  | |  | |  | |  | |  | |  | |  | |  | |  | |  |
| Cardiac history |  | |  | |  | |  | |  | |  | |  | |  | |  | |  | |  | |  |
| No | *Reference* | | - | | - | | - | | - | | - | | - | | - | | - | | - | | - | |  |
| Yes | No | | 1.306*** (.052) | | [1.208 - 1.411] | | 1.226*** (.051) | | [1.130 - 1.331] | | 1.105* (.064) | | [.986 - 1.238] | | 1.275*** (.112) | | [1.074 - 1.514] | | 1.460** (.252) | | [1.040 - 2.048] | |  |
|  |  | |  | |  | |  | |  | |  | |  | |  | |  | |  | |  | |  |
| Heart function class5 |  | |  | |  | |  | |  | |  | |  | |  | |  | |  | |  | |  |
| Class I | *Reference* | | - | | - | | - | | - | | - | | - | | - | | - | | - | | - | |  |
| Class II | No | | .954 (.046) | | [.868 - 1.049] | | .901** (.046) | | [.816 - .996] | | 1.051 (.072) | | [.918 - 1.203] | | 1.151 (.119) | | [.940 - 1.409] | | 1.175 (.237) | | [.792 - 1.744] | |  |
| Class III or IV | No | | .856 (.090) | | [.696 - 1.053} | | .890 (.097) | | [.718 - 1.101] | | 1.068 (.149) | | [.812 - 1.405] | | 1.554** (.271) | | [1.104 - 2.187] | | 1.418 (.462) | | [.749 - 2.685] | |  |
|  |  | |  | |  | |  | |  | |  | |  | |  | |  | |  | |  | |  |
| Respiratory findings |  | |  | |  | |  | |  | |  | |  | |  | |  | |  | |  | |  |
| No dyspnoea | *Reference* | | - | | - | | - | | - | | - | | - | | - | | - | | - | | - | |  |
| Dyspnoea at rest | No | | 1.188 (.546) | | [.482 - 2.925] | | 2.305* (1.051) | | [.943 - 5.633] | | 2.540* (1.275) | | [.950 - 6.791] | | 2.398 (1.383) | | [.774 - 7.426] | | 3.714* (2.952) | | [.782 - 17.640] | |  |
| Dyspnoea limiting exertion | Yes | | 1.404*** (.133) | | [1.166 - 1.691] | | 1.404*** (.133) | | [1.166 - 1.691] | | 1.404*** (.133) | | [1.166 - 1.691] | | 1.404*** (.133) | | [1.166 - 1.691] | | 1.404*** (.133) | | [1.166 - 1.691] | |  |
| Dyspnoea on exertion | Yes | | 1.257*** (.061) | | [1.142 - 1.383] | | 1.257*** (.061) | | [1.142 - 1.383] | | 1.257*** (.061) | | [1.142 - 1.383] | | 1.257*** (.061) | | [1.142 - 1.383] | | 1.257*** (.061) | | [1.142 - 1.383] | |  |
|  |  | |  | |  | |  | |  | |  | |  | |  | |  | |  | |  | |  |
| Variable | Proportional odds upheld? | | Odds ratio CDC>0 vs <=0 (SD) | | 95% conf. interval | | Odds ratio CDC>I vs <=I (SD) | | 95% conf. interval | | Odds ratio CDC>II vs <=II (SD) | | 95% conf. interval | | Odds ratio CDC>III vs <=III (SD) | | 95% conf. interval | | Odds ratio CDC>IV vs <=IV (SD) | | 95% conf. interval | |  |
|  |  | |  | |  | |  | |  | |  | |  | |  | |  | |  | |  | |  |
| Diabetes |  | |  | |  | |  | |  | |  | |  | |  | |  | |  | |  | |  |
| No | *Reference* | | - | | - | | - | | - | | - | | - | | - | | - | | - | | - | |  |
| Type I | Yes | | 1.171 (.212) | | [.822 - 1.670] | | 1.171 (.212) | | [.822 - 1.670] | | 1.171 (.212) | | [.822 - 1.670] | | 1.171 (.212) | | [.822 - 1.670] | | 1.171 (.212) | | [.822 - 1.670] | |  |
| Type II diet controlled only | Yes | | 1.048 (.087) | | [.890 - 1.234] | | 1.048 (.087) | | [.890 - 1.234] | | 1.048 (.087) | | [.890 - 1.234] | | 1.048 (.087) | | [.890 - 1.234] | | 1.048 (.087) | | [.890 - 1.234] | |  |
| Type II on insulin | Yes | | .922 (.079) | | [.780 - 1.090] | | .922 (.079) | | [.780 - 1.090] | | .922 (.079) | | [.780 - 1.090] | | .922 (.079) | | [.780 - 1.090] | | .922 (.079) | | [.780 - 1.090] | |  |
| Type II on non-insulin medication | Yes | | 1.002 (.057) | | [.897 - 1.120] | | 1.002 (.057) | | [.897 - 1.120] | | 1.002 (.057) | | [.897 - 1.120] | | 1.002 (.057) | | [.897 - 1.120] | | 1.002 (.057) | | [.897 - 1.120] | |  |
|  |  | |  | |  | |  | |  | |  | |  | |  | |  | |  | |  | |  |
| Liver disease |  | |  | |  | |  | |  | |  | |  | |  | |  | |  | |  | |  |
| No | *Reference* | | - | | - | | - | | - | | - | | - | | - | | - | | - | | - | |  |
| Yes - cirrhosis or Hep^6^ B/C with portal HTN | Yes | | 1.159 (.355) | | [.635 - 2.114] | | 1.159 (.355) | | [.635 - 2.114] | | 1.159 (.355) | | [.635 - 2.114] | | 1.159 (.355) | | [.635 - 2.114] | | 1.159 (.355) | | [.635 - 2.114] | |  |
| Yes - cirrhosis or Hep B/C without portal HTN | Yes | | 1.303* (.184) | | [.988 - 1.718] | | 1.303* (.184) | | [.988 - 1.718] | | 1.303* (.184) | | [.988 - 1.718] | | 1.303* (.184) | | [.988 - 1.718] | | 1.303* (.184) | | [.988 - 1.718] | |  |
|  |  | |  | |  | |  | |  | |  | |  | |  | |  | |  | |  | |  |
| Smoking history |  | |  | |  | |  | |  | |  | |  | |  | |  | |  | |  | |  |
| Never smoked | *Reference* | | - | | - | | - | | - | | - | | - | | - | | - | | - | | - | |  |
| Current smoker | No | | 1.076 (.059) | | [.966 - 1.199] | | 1.100 (.065) | | [.979 - 1.236] | | 1.131 (.095) | | [.959 - 1.333] | | 1.547*** (.192) | | [1.213 - 1.973] | | 1.230 (.358) | | [.695 - 2.177] | |  |
| Ex-smoker - stopped > 6 months ago | No | | .992 (.035) | | [.925 - 1.064] | | 1.084** (.042) | | [1.005 - 1.168] | | 1.117** (.061) | | [1.004 - 1.242] | | 1.145 (.098) | | [.968 - 1.355] | | 1.118 (.191) | | [.800 - 1.562] | |  |
| Ex-smoker - stopped <= 6 months ago | Yes | | 1.305*** (.093) | | [1.135 - 1.500] | | 1.305*** (.093) | | [1.135 - 1.500] | | 1.305*** (.093) | | [1.135 - 1.500] | | 1.305*** (.093) | | [1.135 - 1.500] | | 1.305*** (.093) | | [1.135 - 1.500] | |  |
| Unknown | Yes | | .806** (.068) | | [.682 - .951] | | .806** (.068) | | [.682 - .951] | | .806** (.068) | | [.682 - .951] | | .806** (.068) | | [.682 - .951] | | .806** (.068) | | [.682 - .951] | |  |
|  |  | |  | |  | |  | |  | |  | |  | |  | |  | |  | |  | |  |
| Alcohol consumption |  | |  | |  | |  | |  | |  | |  | |  | |  | |  | |  | |  |
| No alcohol | *Reference* | | - | | - | | - | | - | | - | | - | | - | | - | | - | | - | |  |
| 0-2 units per day | Yes | | .891*** (.030) | | [.835 - .951] | | .891*** (.030) | | [.835 - .951] | | .891*** (.030) | | [.835 - .951] | | .891*** (.030) | | [.835 - .951] | | .891*** (.030) | | [.835 - .951] | |  |
| 3-4 units per day | Yes | | .895** (.048) | | [.805 - 995] | | .895** (.048) | | [.805 - 995] | | .895** (.048) | | [.805 - 995] | | .895** (.048) | | [.805 - 995] | | .895** (.048) | | [.805 - 995] | |  |
| > 5 units per day | Yes | | 1.063 (.074) | | [.927 - 1.219] | | 1.063 (.074) | | [.927 - 1.219] | | 1.063 (.074) | | [.927 - 1.219] | | 1.063 (.074) | | [.927 - 1.219] | | 1.063 (.074) | | [.927 - 1.219] | |  |
| Unknown | Yes | | .923 (.060) | | [.813 - 1.048] | | .923 (.060) | | [.813 - 1.048] | | .923 (.060) | | [.813 - 1.048] | | .923 (.060) | | [.813 - 1.048] | | .923 (.060) | | [.813 - 1.048] | |  |
| Urgency of surgery |  | |  | |  | |  | |  | |  | |  | |  | |  | |  | |  | |  |
| Elective | *Reference* | | - | | - | | - | | - | | - | | - | | - | | - | | - | | - | |  |
| Expedited | Yes | | 1.095* (.059) | | [.985 - 1.217] | | 1.095* (.059) | | [.985 - 1.217] | | 1.095* (.059) | | [.985 - 1.217] | | 1.095* (.059) | | [.985 - 1.217] | | 1.095* (.059) | | [.985 - 1.217] | |  |
|  |  | |  | |  | |  | |  | |  | |  | |  | |  | |  | |  | |  |
| Variable | | Proportional odds upheld? | | Odds ratio CDC>0 vs <=0 (SD) | | 95% conf. interval | | Odds ratio CDC>I vs <=I (SD) | | 95% conf. interval | | Odds ratio CDC>II vs <=II (SD) | | 95% conf. interval | | Odds ratio CDC>III vs <=III (SD) | | 95% conf. interval | | Odds ratio CDC>IV vs <=IV (SD) | | 95% conf. interval | |
|  | |  | |  | |  | |  | |  | |  | |  | |  | |  | |  | |  | |
| Perioperative risk assessment | |  | |  | |  | |  | |  | |  | |  | |  | |  | |  | |  | |
| No | | *Reference* | | - | | - | | - | | - | | - | | - | | - | | - | | - | | - | |
| Qualitative and Quantitative | | Yes | | .926* (.039) | | [.854 - 1.005] | | .926* (.039) | | [.854 - 1.005] | | .926* (.039) | | [.854 - 1.005] | | .926* (.039) | | [.854 - 1.005] | | .926* (.039) | | [.854 - 1.005] | |
| Qualitative | | Yes | | .910** (.035) | | [.844 - .981] | | .910** (.035) | | [.844 - .981] | | .910** (.035) | | [.844 - .981] | | .910** (.035) | | [.844 - .981] | | .910** (.035) | | [.844 - .981] | |
| Quantitative | | Yes | | 1.084 * (.045) | | [1.000 - 1.175] | | 1.084 * (.045) | | [1.000 - 1.175] | | 1.084 * (.045) | | [1.000 - 1.175] | | 1.084 * (.045) | | [1.000 - 1.175] | | 1.084 * (.045) | | [1.000 - 1.175] | |
|  | |  | |  | |  | |  | |  | |  | |  | |  | |  | |  | |  | |
| Planned surgical specialty | |  | |  | |  | |  | |  | |  | |  | |  | |  | |  | |  | |
| Hepatobiliary | | *Reference* | | - | | - | | - | | - | | - | | - | | - | | - | | - | | - | |
| Abdominal lower gastrointestinal | | No | | .948 (.048) | | [.858 - 1.046] | | .856*** (.045) | | [.772 - .950] | | .932 (.068) | | [.808 - 1.075] | | .993 (.114) | | [.793 - 1.244] | | .737 (.170) | | [.469 - 1.158] | |
| Abdominal upper gastrointestinal | | No | | 1.136** (.074) | | [1.000 - 1.290] | | 1.274*** (.083) | | [1.121 - 1.448] | | 1.405*** (.118) | | [1.193 - 1.656] | | 1.733*** (.213) | | [1.363 - 2.205] | | 1.044 (.269) | | [.630 - 1.731] | |
| Abdominal other | | Yes | | .827** (.065) | | [.710 - .965] | | .827** (.065) | | [.710 - .965] | | .827** (.065) | | [.710 - .965] | | .827** (.065) | | [.710 - .965] | | .827** (.065) | | [.710 - .965] | |
|  | |  | |  | |  | |  | |  | |  | |  | |  | |  | |  | |  | |
| Post-operative destination | |  | |  | |  | |  | |  | |  | |  | |  | |  | |  | |  | |
| Ward care | | *Reference* | | - | | - | | - | | - | | - | | - | | - | | - | | - | | - | |
| Level 1 or enhanced care | | No | | 1.123** (.056) | | [1.019 - 1.238] | | 1.284*** (.073) | | [1.150 - 1.435] | | 1.103 (.101) | | [.923 - 1.319] | | .978 (.183) | | [.678 - 1.411] | | 1.147 (.380) | | [.599 - 2.197] | |
| Level 2 care | | No | | 1.681*** (.065) | | [1.559 - 1.814] | | 1.921*** (.083) | | [1.765 - 2.091] | | 1.868*** (.121) | | [1.645 - 2.121] | | 2.496*** (.299) | | [1.974 - 3.156] | | 2.404*** (.569) | | [1.512 - 3.823] | |
| Level 3 care | | No | | 3.848*** (.278) | | [3.340 - 4.433] | | 4.589*** (.324) | | [3.995 - 5.270] | | 3.673*** (.330) | | [3.080 - 4.381] | | 6.721*** (.941) | | [5.107 - 8.844] | | 5.047 *** (1.492) | | [2.828 - 9.007] | |
|  | |  | |  | |  | |  | |  | |  | |  | |  | |  | |  | |  | |
| Employment status | |  | |  | |  | |  | |  | |  | |  | |  | |  | |  | |  | |
| Employed | | *Reference* | | - | | - | | - | | - | | - | | - | | - | | - | | - | | - | |
| Parent or carer | | Yes | | .975 (.104) | | [.792 - 1.201] | | .975 (.104) | | [.792 - 1.201] | | .975 (.104) | | [.792 - 1.201] | | .975 (.104) | | [.792 - 1.201] | | .975 (.104) | | [.792 - 1.201] | |
| Retired | | Yes | | 1.060 (.046) | | [.974 - 1.154] | | 1.060 (.046) | | [.974 - 1.154] | | 1.060 (.046) | | [.974 - 1.154] | | 1.060 (.046) | | [.974 - 1.154] | | 1.060 (.046) | | [.974 - 1.154] | |
| Unemployed | | Yes | | 1.218* (.135) | | [.980 - 1.514] | | 1.218* (.135) | | [.980 - 1.514] | | 1.218* (.135) | | [.980 - 1.514] | | 1.218* (.135) | | [.980 - 1.514] | | 1.218* (.135) | | [.980 - 1.514] | |
| Unemployed due to health reasons | | Yes | | 1.419***(.094) | | [1.248 - 1.615] | | 1.419***(.094) | | [1.248 - 1.615] | | 1.419***(.094) | | [1.248 - 1.615] | | 1.419***(.094) | | [1.248 - 1.615] | | 1.419***(.094) | | [1.248 - 1.615] | |
| Unavailable | | No | | 1.054 (.070) | | [.925 - 1.201] | | 1.048 (.076) | | [.909 - 1.208] | | 1.063 (.110) | | [.867 - 1.303] | | 1.320* (.200) | | [.981 - 1.777] | | 2.355*** (.598) | | [1.431 - 3.875] | |

^1^ Body mass index

^2^ American Society of Anaesthesiologists physical status classification

^3^ Electrocardiogram

^4^ Includes any other abnormal rhythm/paced rhythm/>5VE/min/Q ST or T wave abnormalities

^5^ New York Heart Association (NYHA) functional classification

^6^ Hepatitis

**Table S3. Data completeness at three timepoints**

| EQ-5D-5L data | Complete (%) | Missing (%) |
| --- | --- | --- |
| Admission | 18,080 (91.8) | 1,605 (8.2) |
| 6 month follow up | 10,229 (52.0) | 9,456 (48.0) |
| 12 month follow up | 8,864 (45.0) | 10,821 (55.0) |
| Sample size n = 19,685 |  |  |

**Table S4. Patterns of EQ-5D-5L missingness**

| Frequency (%) | Admission | 6 month follow up | 12 month follow up |
| --- | --- | --- | --- |
| 6,717 (34.1) | 1 | 1 | 1 |
| 7,043 (35.8) | 1 | 0 | 0 |
| 2,791 (14.2) | 1 | 1 | 0 |
| 1,529 (7.8) | 1 | 0 | 1 |
| 752 (3.8) | 0 | 0 | 0 |
| 486 (2.5) | 0 | 1 | 1 |
| 235 (1.2) | 0 | 1 | 0 |
| 132 (0.7) | 0 | 0 | 1 |
| Sample size n = 19,685  1 = complete  0 = missing |  |  |  |

**Table S5. Tobit, OLS, and multiple imputation regression results.**

| Variable | Univariate Tobit | Univariate OLS | Multivariate Tobit | | Multivariate OLS | | OLS Multiple imputation | |
| --- | --- | --- | --- | --- | --- | --- | --- | --- |
|  |  |  |  | |  | |  | |
| **Clavien-Dindo grade** |  |  |  | |  | |  | |
| Grade 0 | *Reference* | - | - | | - | | - | |
| Grade I | -.030*** (.006) | -.029*** (.006) | -.013*** (.005) | | -.012** (.005) | | -.011** (.004) | |
| Grade II | -.058*** (.006) | -.055*** (.006) | -.028*** (.005) | | -.026*** (.005) | | -.025*** (.005) | |
| Grade III | -.075*** (.010) | -.073*** (.010) | -.034*** (.007) | | -.033*** (.007) | | -.033*** (.008) | |
| Grade IV | -.150*** (.018) | -.146*** (.017) | -.089*** (.014) | | -.086*** (.014) | | -.085*** (.012) | |
| Grade V | -.818*** (.003) | -.811*** (.003) | -.757*** (.012) | | -.750*** (.012) | | -.749*** (.012) | |
| **Age** | .001*** (.000) | .001*** (.000) | .000 (.000) | | -.000 (.000) | | -.000 (.000) | |
| **Gender** |  |  |  | |  | |  | |
| Female | *Reference* | - | - | | - | | - | |
| Male | .037*** (.005) | .026*** (.005) | .005 (.003) | | -.003 (.003) | | -.003 (.003) | |
| **ASA** |  |  |  | |  | |  | |
| Class I | .035*** (.007) | .036*** (.007) | .013*** (.005) | | .013*** (.005) | | .011** (.005) | |
| Class II | *Reference* | - | - | | - | | - | |
| Class III | -.096*** (.006) | -.093*** (.006) | -.027*** (.005) | | -.025*** (.004) | | -.025*** (.004) | |
| Class IV or V | -.223*** (.031) | -.217*** (.031) | -.058** (.024) | | -.055** (.024) | | -.054*** (.019) | |
| **Heart function class** |  |  |  | |  | |  | |
| Class I | *Reference* | - | - | | - | | - | |
| Class II | -.057*** (.008) | -.055*** (.007) | -.004 (.005) | | -.002 (.005) | | -.003 (.005) | |
| Class III or IV | -.200*** (.021) | -.196*** (.020) | -.023 (.014) | | -.024* (.014) | | -.027** (.012) | |
| **Respiratory findings** |  |  |  | |  | |  | |
| No dyspnoea | *Reference* | - | - | | - | | - | |
| Dyspnoea at rest | -.242** (.120) | -.235* (.120) | .019 (.082) | | .021 (.080) | | .021 (.059) | |
| Dyspnoea limiting exertion | -.202*** (.020) | -.198*** (.020) | -.023* (.014) | | -.024* (.013) | | -.022* (.013) | |
| Dyspnoea on exertion | -.089*** (.009) | -.086*** (.009) | -.019*** (.006) | | -.018*** (.006) | | -.019*** (.005) | |
| **Diabetes** |  |  |  | |  | |  | |
| No | *Reference* | - | - | | - | | - | |
| Type I | .017 (.028) | .019 (.027) | .026 (.020) | | .028 (.019) | | .027 (.018) | |
| Type II - diet controlled only | -.034** (.014) | -.032** (.014) | -.013 (.010) | | -.010 (.009) | | -.011 (.008) | |
| Type II - on insulin | -.089*** (.017) | -.088*** (.017) | -.033*** (.012) | | -.032*** (.012) | | -.035*** (.011) | |
| Type II - on non-insulin medication | -.045*** (.011) | -.045*** (.010) | -.023*** (.008) | | -.021*** (.007) | | -.021*** (.007) | |
| Variable | Univariate Tobit | Univariate OLS | Multivariate Tobit | Multivariate OLS | | OLS Multiple imputation | |  |
| **Smoking history** |  |  |  |  | |  | |  |
| Never smoked | - | - | - | - | | - | |  |
| Current smoker | -.105*** (.010) | -.101*** (.010) | -.031*** (.008) | -.031*** (.007) | | -.032*** (.006) | |  |
| Ex-smoker- stopped > 6 months ago | -.023*** (.006) | -.024*** (.005) | -.007* (.004) | -.007* (.004) | | -.006* (.003) | |  |
| Ex-smoker- stopped <= 6 months ago | -.075*** (.015) | -.074*** (.014) | -.025*** (.010) | -.025*** (.009) | | -.025*** (.009) | |  |
| Unknown | -.016 (.013) | -.017 (.013) | -.011 (.010) | -.011 (.010) | | -.012 (.009) | |  |
| **Alcohol consumption** |  |  |  |  | |  | |  |
| No alcohol | - | - | - | - | | - | |  |
| 0-2 units per day | .054*** (.006) | .051*** (.006) | .010*** (.004) | .010*** (.004) | | .010** (.004) | |  |
| 3-4 units per day | .059*** (.009) | .054*** (.008) | .007 (.006) | .007 (.006) | | .006 (.005) | |  |
| > 5 units per day | .047*** (.012) | .041*** (.011) | .013* (.008) | .012* (.007) | | .013 (.008) | |  |
| Unknown | .024 ** (.011) | .022** (.011) | .007 (.008) | .006 (.008) | | .005 (.008) | |  |
| **Admission EQ-5D-3L index value** | .727*** (.012) | .692*** (.012) | .660*** (.011) | .637*** (.011) | | .635*** (.009) | |  |
| **Planned surgical specialty** |  |  |  |  | |  | |  |
| Hepatobiliary | - | - | - | - | | - | |  |
| Abdominal lower gastrointestinal | .062*** (.008) | .059*** (.008) | .048*** (.006) | .047*** (.006) | | .046*** (.005) | |  |
| Abdominal upper gastrointestinal | -.008 (.012) | -.008 (.012) | -.008 (.009) | -.006 (.008) | | -.007 (.008) | |  |
| Abdominal other | .017 (.015) | .018 (.015) | .050*** (.009) | .048*** (.009) | | .047*** (.009) | |  |
| **Employment status** |  |  |  |  | |  | |  |
| Employed | - | - | - | - | | - | |  |
| Parent or carer | -.091*** (.018) | -.090*** (.017) | -.023* (.012) | -.027* (.012) | | -.025* (.014) | |  |
| Retired | -.016*** (.005) | -.022*** (.005) | .007 (.005) | .005 (.005) | | .004 (.004) | |  |
| Unemployed | -.078*** (.021) | -.075*** (.020) | -.026* (.015) | -.027* (.015) | | -.028** (.012) | |  |
| Unemployed due to health | -.237*** (.015) | -.235*** (.015) | -.064*** (.010) | -.069*** (.010) | | -.072*** (.009) | |  |
| Unavailable | -.408*** (.072) | -.407*** (.071) | .030 (.032) | .027 (.031) | | .024 (.030) | |  |
| **Constant** |  |  | .242*** (.015) | .275*** (.014) | | .277*** (.013) | |  |
|  |  |  |  |  | |  | |  |
| Number of observations | - | - | 8,693 | 8693 | | 16514 | |  |

**Table S6. English NHS morbidity QALY loss due to postoperative complications in 2018/2019.**

| Clavien-Dindo grade | Study sample prevalence | Estimated nationwide frequency | Multivariable OLS coefficient | QALY loss |
| --- | --- | --- | --- | --- |
|  |  |  |  |  |
| I | 16.1% | 43,375 | -0.012 | -514 |
| II | 18.6% | 50,015 | -0.026 | -1,302 |
| III | 7.1% | 19,013 | -0.033 | -633 |
| IV | 2.8% | 7,421 | -0.086 | -635 |
|  |  |  |  |  |
| Total |  |  |  | -3,083 |
